# Quantitative Imaging of Blood-Brain Barrier Permeability Following Repetitive Mild Head Impacts

**DOI:** 10.3389/fneur.2021.729464

**Published:** 2021-09-30

**Authors:** Joshua Leaston, Ju Qiao, Ian C. Harding, Praveen Kulkarni, Codi Gharagouzloo, Eno Ebong, Craig F. Ferris

**Affiliations:** ^1^Imaginostics, Inc., Cambridge, MA, United States; ^2^Center for Translational Neuroimaging, Northeastern University, Boston, MA, United States; ^3^Department of Bioengineering, Northeastern University, Boston, MA, United States; ^4^Department of Chemical Engineering, Northeastern University, Boston, MA, United States; ^5^Departments of Psychology and Pharmaceutical Sciences, Northeastern University, Boston, MA, United States

**Keywords:** concussion, ferumoxytol, cerebral small vessel disease, momentum exchange, quantitative ultrashort time-to-echo, contrast enhanced MRI

## Abstract

This was an exploratory study designed to evaluate the feasibility of a recently established imaging modality, quantitative ultrashort time-to-echo contrast enhanced (QUTE-CE), to follow the early pathology and vulnerability of the blood brain barrier in response to single and repetitive mild head impacts. A closed-head, momentum exchange model was used to produce three consecutive mild head impacts aimed at the forebrain separated by 24 h each. Animals were measured at baseline and within 1 h of impact. Anatomical images were collected to assess the extent of structural damage. QUTE-CE biomarkers for BBB permeability were calculated on 420,000 voxels in the brain and were registered to a bilateral 3D brain atlas providing site-specific information on 118 anatomical regions. Blood brain barrier permeability was confirmed by extravasation of labeled dextran. All head impacts occurred in the absence of any structural brain damage. A single mild head impact had measurable effects on blood brain barrier permeability and was more significant after the second and third impacts. Affected regions included the prefrontal ctx, basal ganglia, hippocampus, amygdala, and brainstem. Our findings support the concerns raised by the healthcare community regarding mild head injuries in participants in organized contact sports and military personnel in basic training and combat.

## Introduction

There is a growing concern and expanding literature on the behavioral and neurobiological consequences of repetitive mild head impacts or concussions incurred while playing organized sports or in military combat. Mild head impacts are estimated to account for 75% of all traumatic brain injuries (1). Concussion following a single incident is difficult to detect and any associated cognitive and behavioral problems can resolve within hours of insults (2, 3). However, a more pernicious, long-lasting condition arises when the brain is exposed to repeated mild head impacts (4, 5). Repetitive head impacts induce cognitive, motor and behavioral deficits, which are more severe and protracted, and can last for months and even years (6, 7) with an increased risk of dementia, and chronic traumatic encephalopathy (CTE) (8, 9).

Failure in the blood brain barrier (BBB) lies at the foundation of cerebrovascular dysfunction as first described by Wardlaw (10). BBB failure is characterized by hyperpermeability of endothelial walls, damage to basement membranes, and enlargement of surrounding perivascular space allowing protein, macrophage, and lymphocyte invasion and β-amyloid (Aβ) deposition (11, 12). Disruption in the BBB commonly occurs with moderate to severe traumatic brain injury (TBI) (13–17) and the insult may persist for years contributing to the neuropathology of neurodegenerative diseases (18). Animal models of repetitive mild head impact report no effect on BBB permeability (19, 20), or a modest increase that persists up to 3 days post insult (21, 22). Additionally, it has been documented in literarture that an early response to TBI may be a decrease in cerebral blood flow (23, 24), and it is speculated that this may play an important role in inhibiting the recovery process of repetitive mild TBI (rmTBI).

There are multiple imaging protocols for detecting the gross lesions that result from the neuropathological consequences of cerebral vascular injury, such as T2 Fluid Attenuated Inversion Recovery (FLAIR), Susceptibility Weighted Imaging (SWI), and Diffusion-Weighted Imaging (DWI). However, these methods cannot quantitatively assess BBB integrity (25). The most common way for assessing BBB leakage is dynamic contrast enhanced (DCE) MRI with gadolinium-based contrast agents (GBCAs) (26), particularly with respect to BBB permeability following head injury (27). DCE-MRI is limited in error in the arterial input function (AIF) (28, 29) and significant variances are reported for rates of leakage (30). More recently, higher accuracy measurements have been achieved in rodents (31, 32) and humans (33, 34), however DCE-MRI remains limited to 2-dimensional imaging with thick slabs (1 mm in mice, 5 mm in humans)—in addition to requiring toxic GBCAs which have recently obtained an FDA black-box warning for brain retention in 2017. To address the need for safe, quantitative, whole-brain non-invasive precision medicine diagnostics for mild brain injury, we explored the use of a recently established alternative technique quantitative ultrashort time-to-echo contrast enhanced (QUTE-CE) MRI (35–39). This method has recently been utilized to map BBB leakage due to Type-2 Diabetes (38). Here, for the first time, we report the use of this technique to measure BBB leakage at the individual animal level, and for head injury, in a model of repeated mild impacts.

## Materials and Methods

### Animals

Subjects were all adult male Sprague Dawley rats (*n* = 5), ~100 days of age and purchased from Charles River Laboratories (Wilmington, MA, USA). Animals were housed in Plexiglas cages and maintained in ambient temperature (22–24°C) on a 12:12 light-dark cycle (lights on at 07:00 a.m.). Food and water were provided *ad libitum*. All methods and procedures described were approved by the Northeastern University Institutional Animal Care and Use Committee (IACUC). The Northeastern facility is AAALAC accredited with OLAW Assurance and is registered with the USDA. All housing, care, and use followed the Guide for the Care and Use of Laboratory Animals (8th Addition) and the Animal Welfare Act. The protocols used in this study adhere to the ARRIVE guidelines for reporting *in vivo* experiments in animal research (40).

### Momentum Exchange Model

Concussion were generated with a pneumatic pressure drive, 50 g compactor described by Viano et al. (41) and refined by Mychasiuk et al. (42) to reliably produce the 7.4 m/s impact velocities described for mild rat head injury. The kinetic energy at impact is 1.37 joules. We have used this model to publish on the long-term neuroradiological effects of repetitive mild head impacts in rats (43). The impact piston was directed to the top of the skull, midline, in the approximate area of Bregma (Supplementary Figure 2A) while rats were anesthetized under 1–2% isoflurane. Rats were awake and ambulatory within 5–7 min after anesthesia and concussion.

### Imaging

Studies were done on a Bruker Biospec 7.0T/20-cm USR horizontal magnet (Bruker, Billerica, MA, USA) and a 20-G/cm magnetic field gradient insert (ID = 12 cm) capable of a 120-μs rise time. Radio frequency signals were sent and received with a quadrature volume coil built into the rat restrainer (Ekam Imaging, Boston, MA, US). All rats imaged under 1–2% isoflurane while keeping a respiratory rate of 40–50 breadths/min.

Animals were measured for baseline prior to being subjected to any head impact. Beginning 24 h later, each rat was subjected to three mild head impacts separated by 24 h intervals as previously described (43). All rats were imaged within 1 h of head impact. Following the final impact, rats were anesthetized with isoflurane exposure and transcardially perfused for postmortem histology.

At the beginning of each imaging session, a high-resolution anatomical data set was collected using the RARE pulse sequence with following parameters, 35 slice of 0.7 mm thickness; field of view [FOV] 3 cm; 256 × 256; repetition time [TR] 3,900 ms; effective echo time [TE] 48 ms; number of excitations (NEX) 3; 6 min 14 s acquisition time. Rats were imaged prior to and following an i.v. bolus of 6 mg/ml Fe of Ferumoxytol. The injected volume was tailored for each rat (assuming 7% blood by body weight) to produce a starting blood concentration of 200 μg/ml Fe (twice the clinical dose approved for use in humans). 3D-UTE image acquisition parameters were TE = 13 μs, TR = 4 ms, and flip angle (FA) = 20° with a RF hard pulse bandwidth of 200 kHz. Therefore, the pulse duration was short (6.4 μs) compared to the T2 of the approximate ferumoxytol concentration (4.4 ms for 3.58 mM, i.e., 200 μg/ml) to minimize signal blur and reduce the probability for a curved trajectory of the magnetization vector Mz. A 3 × 3 × 3 cm^3^ field-of-view was used with a matrix mesh size of 180 × 180 × 180 to produce 167 μm isotropic resolution. Example images from scan sessions can be seen in Supplementary Figure 1.

### Image Processing

Images were motion-corrected, aligned spatially, and resliced with nearest neighbor algorithms to preserve original intensity values using MATLAB SPM12 toolbox develop at UCL (http://www.fil.ion.ucl.ac.uk/spm/). The T2-weighted RARE anatomical data for each rat taken at each imaging session was fit to a 3D MRI Rat Brain Atlas © (Ekam Solutions LLC, Boston, MA, US) with 118 segmented annotated brain areas using software developed at Northeastern University's Center for Translational Neuroimaging (CTNI). The fitted atlas was used for region-of-interest (ROI) selection to determine site-specific abnormality. Image intensity was corrected for B1^−^ coil sensitivity along the z-axis using a homogenous copper sulfate 10-ml tube phantom. Anatomical description of atlas regions by cluster can be found in Supplementary Table 1.

### BBB Permeability Biomarker

In this experimental design, quantification of the BBB permeability was achieved by analyzing the slope of the CBV vs. time curve over 7 consecutive post-contrast scans (see Supplementary Figure 2B). Fluctuations in CBV in each region of interest was attributed to modulations in BBB permeability, and were calculated by the percentage change in apparent CBV per second.

### Statistical Analysis

Group analyses were conducted with one-tailed repeated measure Analysis of Variance (rmANOVA), followed by several planned pairwise comparisons to assess the effect of repetitive mild head impacts on each brain region. Using this model, all subsequent scanning sessions were statistically compared to baseline to isolate the effect of head impacts on the BBB. To control for the effect of both type-I error and type-II error, multiple comparisons correction was applied through the Benjamin-Hochberg procedure with a false discovery rate (*p* < 0.05 & FDR = 0.1) for each family of hypotheses. Random missing data was accounted for with mean imputation.

To determine if increases in BBB permeability could be detected at the individual animal level, the slope of each animal's CBV was analyzed over 7 scans with one-tailed linear regression (*p* < 0.1). This analysis was accompanied by Bonferroni correction predicated on the number of statistical tests in each data sample.

Blood brain barrier permeability (BBB) quantification were analyzed by the fitment of an atlas with 118 regions of interest. All significant individual animal results were assessed daily, and group averages were calculated to compare the BBB of each subsequent day to the respective control values.

All analyses were performed in MATLAB, SPSS version 27, and GraphPad Prism.

### Histological Analysis

Changes in BBB permeability following mild impacts was confirmed via injection of FITC-fluorescent dextran, as previously described (44). Briefly, 70 kDa FITC-dextran was injected intravenously to rats immediately after imaging. One hour after injection, rats were perfused and their brains collected, post fixed in 4% PFA for 24 h, cryopreserved in 30% sucrose for 48 h, and subsequently sectioned in 40-micron increments. Sections were stained with *Lycopersicon esculentum* (Tomato) lectin to label the vasculature and imaged using a laser scanning confocal microscope. Increased FITC signal in the perivascular space was indicative of increased BBB permeability. Permeability was quantified by analyzing mean fluorescent intensity of FITC-dextran outside of the vasculature (44). Images were taken using a Zeiss LSM 880 laser scanning confocal microscope in Northeastern University's Institute for Chemical Imaging of Living Systems. Regions found with increased BBB permeability with QUTE-CE imaging (orbital and motor cortices) were compared to areas that demonstrated no significant permeability differences (substantia nigra).

### Data Availability

Data for average CBV values for all animals, days, scans, and region can be accessed at https://github.com/Gharagouzloo/FrontiersinNeurology2021.

## Results

### Structural Results

**Figure 1:**
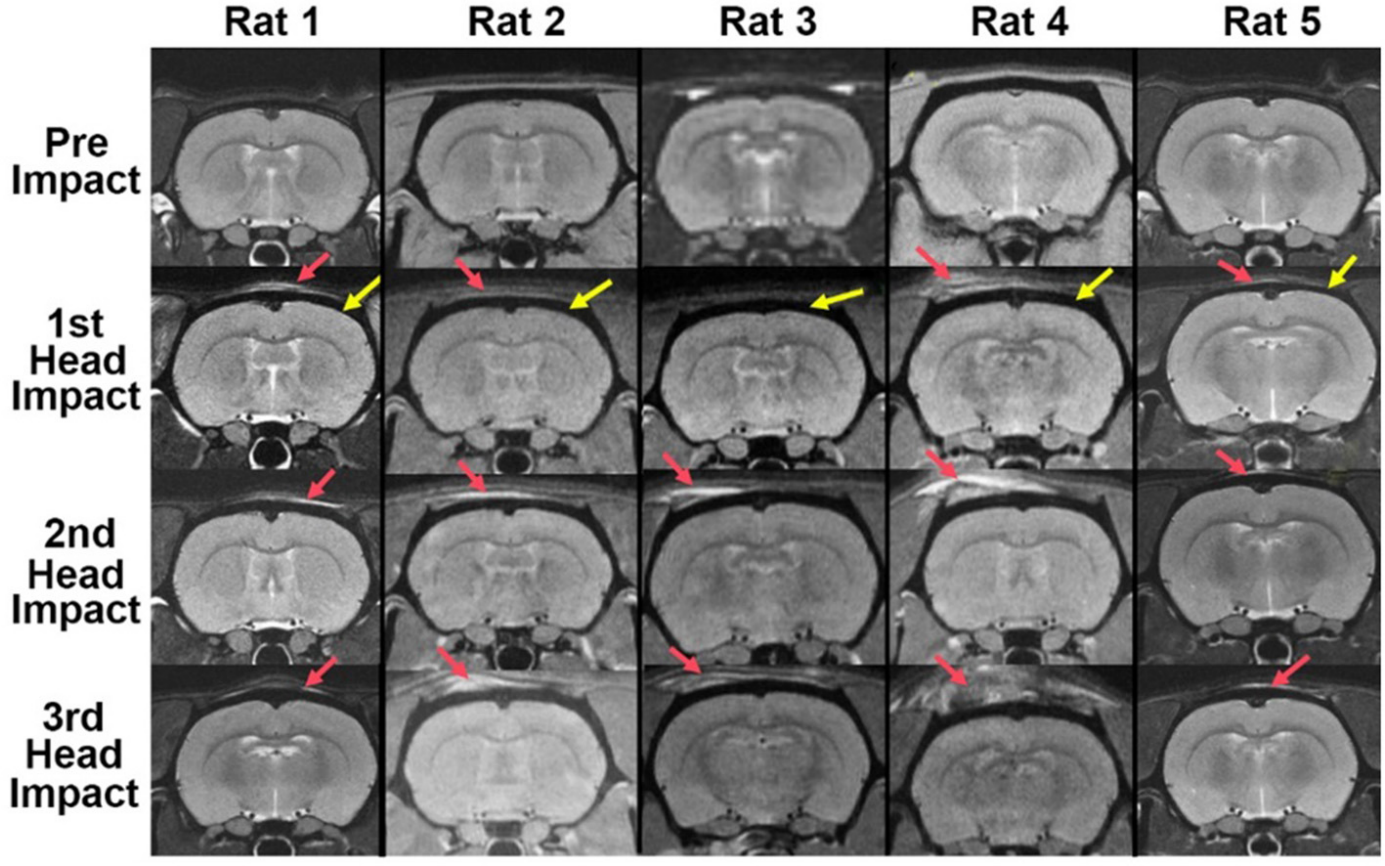
Brain neuroanatomy following repetitive mild head impacts. Mild head impact without evidence of brain damage. Shown are axial sections of T2-weighted anatomical images taken within 1 h of mild head impact. The site of impact noted by the red arrow can be identified by the high intensity signal reflecting the edema on the tissue. The yellow arrows point to the black contour of the cranium. There is no visible evidence of structural brain damage.

**Figure 2:**
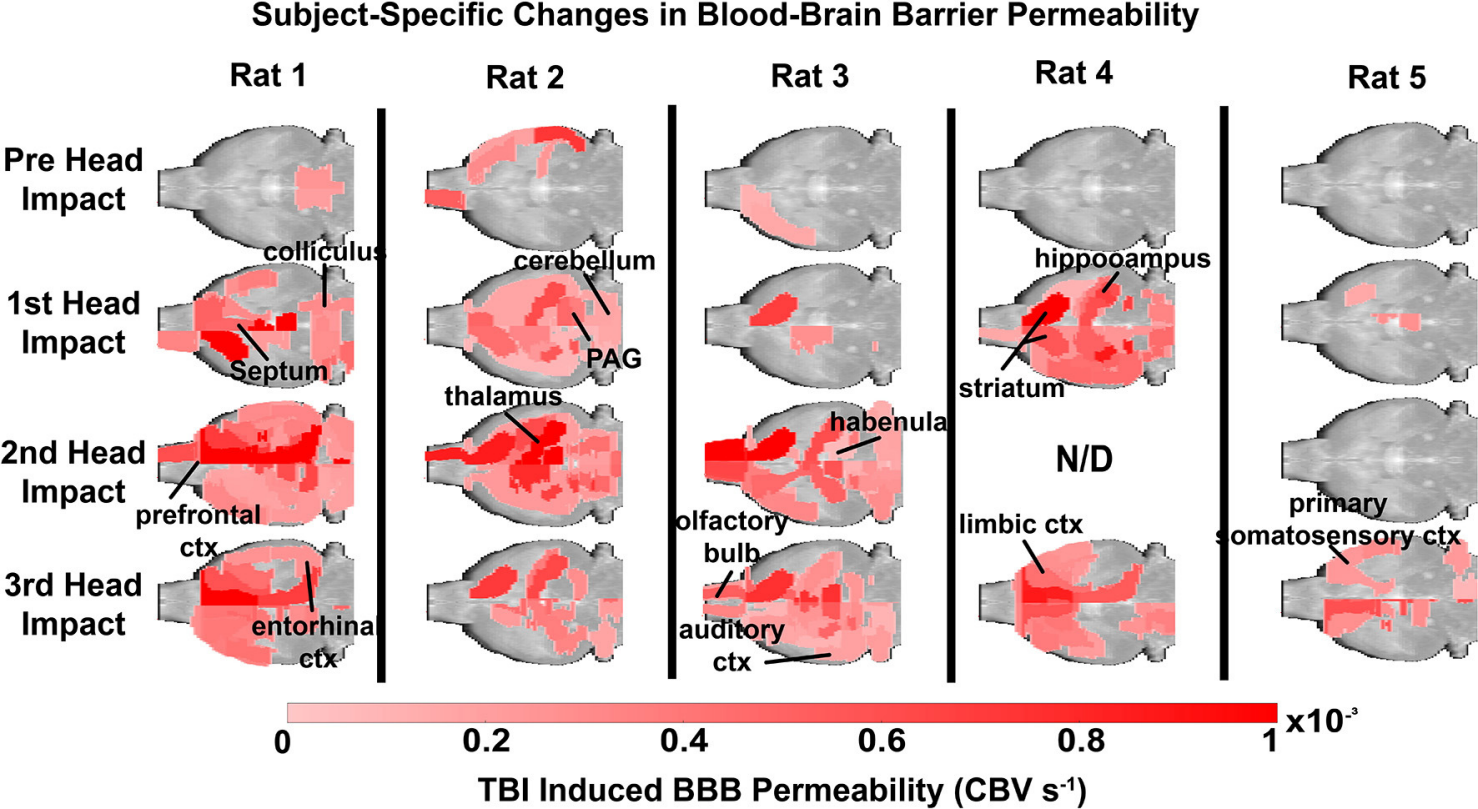
Subject site-specific changes in blood brain barrier permeability. Each animal was scanned at baseline and following mild head impacts (1/day). Significance was determined by a linear regression model to assess the slope over the course of each imaging session, and then adjusted with a Bonferroni correction. Notably, increased BBB permeability was found near the site of impact. The color scale represents the slope and is only displayed if significant (*p* < 0.1, FDR = 0.1).

There was no evidence of structural brain damage after mild head impact at any of the time points for any of the rats (Figure 1). The approximate site of the impact (red arrow) can be identified by the high intensity signal reflecting the edema on the tissue above the skull (black encasement of brain denoted by the yellow arrow). While the edema is apparent in most of the images, it is not obvious in Rat 3 after the 1st mild head impact and only slightly so in Rat 2 and Rat 5. Nonetheless, Rat 2 showed a diffuse pattern of increased brain permeability after the 1st impact while Rats 3 and 5 showed very little (see Figure 2).

### Subject Site-Specific Changes in Blood Brain Permeability

**Figure 3:**
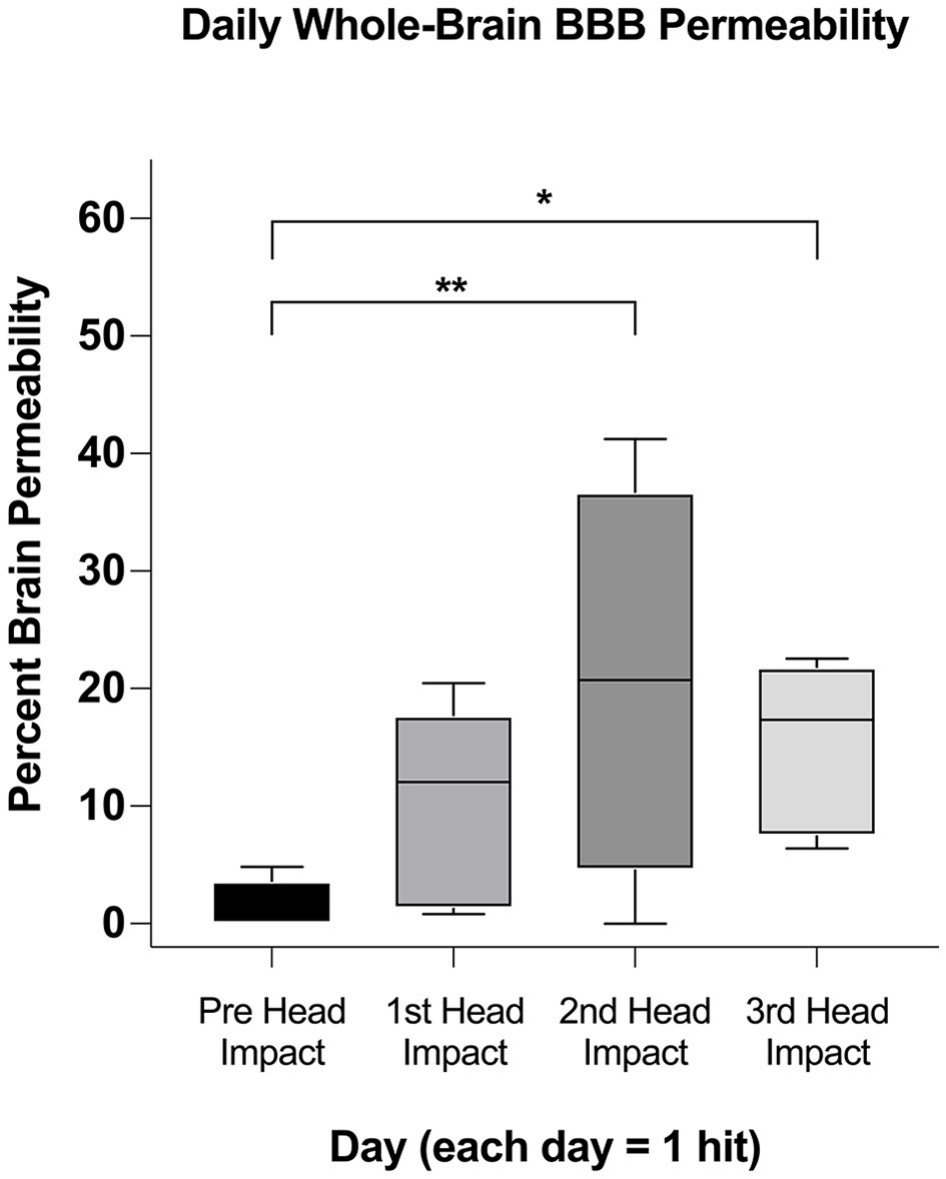
Whole-brain comparison of percent brain volume affected in subjects. After ascertaining if a region was affected in each animal (Figure 2) we used a one-tailed mixed effect analysis (*p* < 0.05; FDR = 0.1) and Benjamin Hochberg correction to determine if the affected brain volumes changed between days. Significance in this figure is represented by asterisks (*p < 0.05, **p < 0.01).

Figure 2 depicts heat maps showing the location of increases in BBB permeability for all animals (columns) prior to and following head impacts (rows). Data was acquired 1 h after each mild head impact. Data is missing (N/D) from Rat 4 from the 2nd impact because of technical issues. The coronal sections enable the visualization of the lateralization of BBB permeability changes (e.g., Rat 1: prefrontal, anterior, and retrosplenial cortices; Rat 3: striatum). The variability within and between subjects over the three impacts is also displayed. For example, Rats 1, 2, and 4, present with increased BBB permeability after the first impact, while Rats 3 and 5 do not. Indeed, Rat 5 appears resilient to the repetitive impacts, only showing a modest increase in BBB permeability in the somatosensory cortex and prefrontal cortex after the 3rd impact (see Supplementary Table 1, Supplementary Figure 3). The box and whisker plot shows the average permeability across rows for each condition as a percentage of the whole brain. Baseline permeability without impact was less than 1% of the total brain permeability (Figure 3). The greatest significant increase in permeability occurred after the 2nd and 3rd impacts as compared to no impact.

### Group Site-Specific Changes in Blood Brain Barrier Permeability

**Figure 4:**
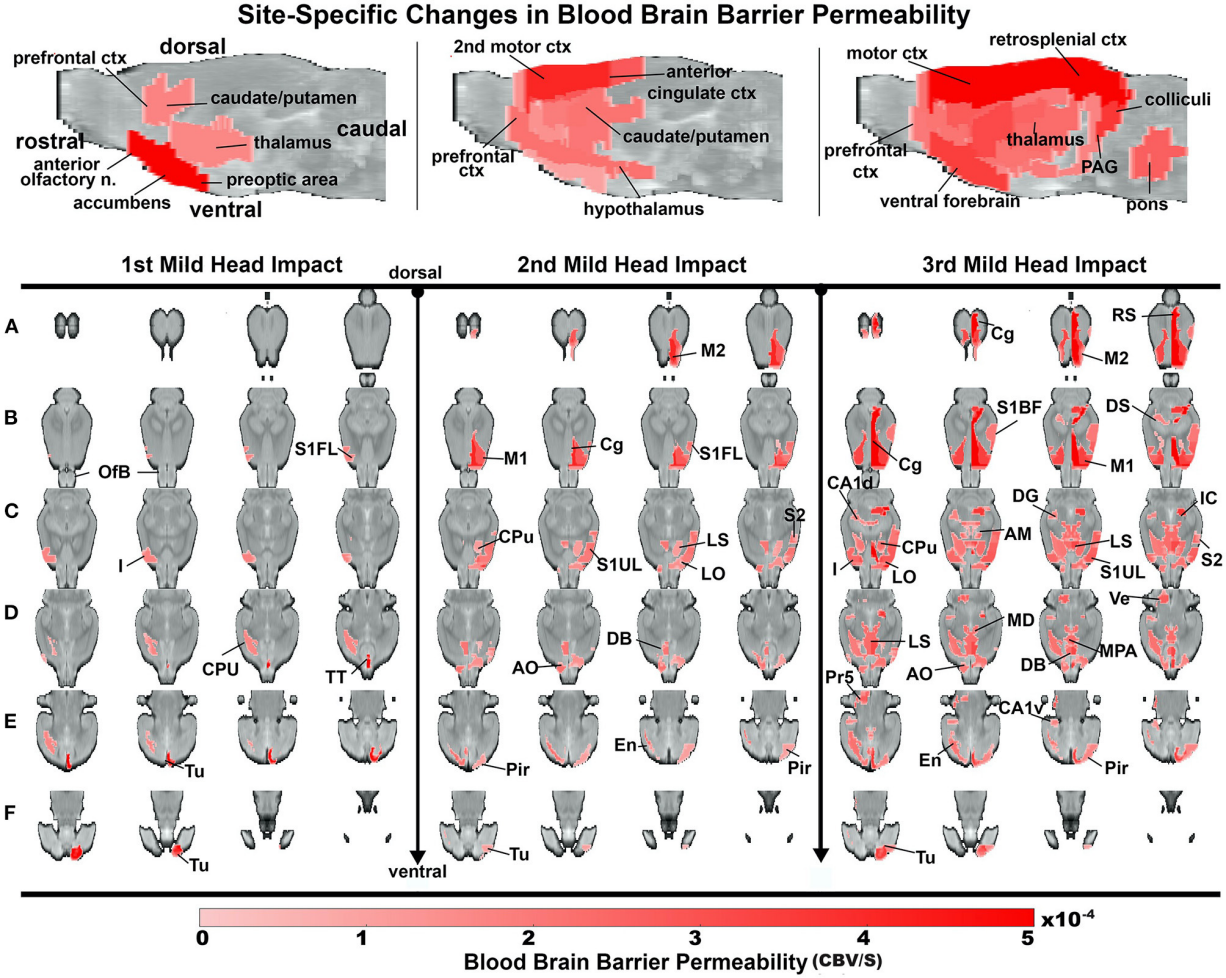
Group site-specific changes in BBB permeability. A one-tailed repeated measures ANOVA with a Benjamin Hochberg correction (*p* < 0.05; FDR = 0.1) was used to compare days of impact to the baseline. Coronal slices of the rat brain from dorsal to ventral depict regions of increased BBB permeability. Notably, the site of impact tested positive after the 2nd and 3rd impact. The color scale value is the difference in slope from baseline and is only displayed if the region is statistically significant.

Heat maps depicting the site-specific location of the average increase in BBB permeability for all subjects are shown in Figure 4. The sagittal sections above show the progressive increase in permeability with repetitive mild head impacts starting in the forebrain with the prefrontal cortex and anterior olfactory tubercles and extending caudally to the retrosplenial ctx, colliculi, and pons. The coronal sections aligned dorsal (top) to ventral (bottom) below show the lateralization and exacerbated permeability with repeated head impacts as exemplified by the 2nd mild head impact (e.g., row b. M2, M1, CG, S1UL, and S2). Table 1 shows all the areas that have a significant increase in BBB permeability for each of the mild head impacts and their lateralization to either the right or left side of the brain. All the head impacts include increased BBB permeability in the olfactory system including the olfactory tubercles (Tu), tenia tecta (TT), piriform ctx (Pir), endopiriform ctx (En), and anterior olfactory nucleus (AON). The caudate putamen (CPu) or striatum is also affected with each mild head impact. With the 3rd mild impact, the increased permeability appears in hindbrain areas that include the hippocampus (row c. DG -dentate gyrus, row b. DS—dorsal subiculum, row c. CA1d, e. CA1v), inferior colliculus (IC), principle trigeminal nucleus (Pr5), and vestibular nucleus (Ve). Progressive BBB permeability is observed from Day 1 at 2%, Day 2 at 7% and Day 3 at 19% whole-brain BBB permeability (Supplementary Figure 4, Supplementary Table 2).

**Table 1:** Site-specific changes in blood brain barrier permeability with repetitive mild head impacts.

	**Right brain**			**Left brain**		
**Brain area**	**1st hit**	**2nd hit**	**3rd hit**	**1st hit**	**2nd hit**	**3rd hit**
**CHANGE IN BLOOD BRAIN BARRIER PERMEABILITY**						
Thalamus			0.012			0.012
CA1 hippocampus						0.031
Anterior cingulate			0.023			
Medial striatum caudate/putamen			0.023		0.031	0.040
Anterior olfactory n., endopiriform		0.002	0.025			
Septum		0.047	0.050			0.026
2nd motor ctx			0.007		0.007	0.004
Primary motor ctx					0.019	0.015
Prefrontal ctx					0.011	0.010
Deep cerebellar n.			0.007			
Rostral piriform ctx					0.027	0.039
Lateral striatum, caudate/putamen	0.038		0.045			
Subiculum hippocampus			0.023			
Inferior colliculus						0.033
Retrosplenial ctx						0.012
Somatosensory ctx barrel field						0.046
Somatosensory ctx jaw	0.021		0.029		0.030	0.024
Somatosensory ctx upper lip					0.022	0.018
Olfactory tubercles, tenia tecta				0.019		0.037

*Shown are the p-values for the average change in BBB to the right or left side of the brain for specific brain areas following one, two, or three minor head impacts (hits)*.

### Histology

**Figure 5:**
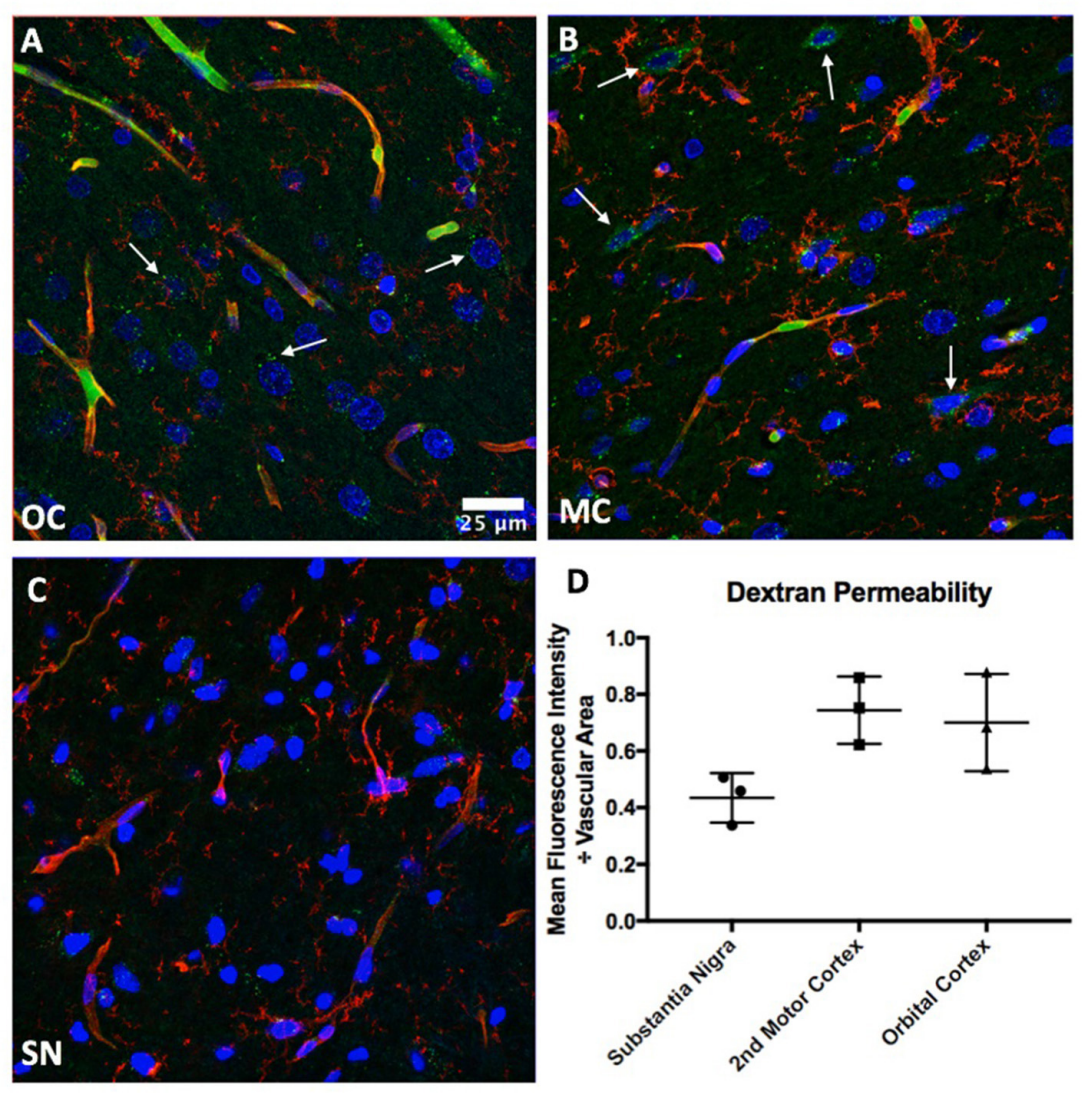
Histological validation of BBB permeability. Fluorescent staining of the vasculature via Tomato lectin (red) demonstrates increases in FITC-dextran (green) accumulation within the parenchyma and vascular cells of the **(A)** orbital (OC) and **(B)** motor (MC) cortices, but not the **(C)** substantia nigra (SN). **(D)** Quantification of permeability. Nuclei are labeled in blue.

Brain regions with increased permeability as identified by QUTE-CE were validated via FITC-dextran injection (Figure 5). Fluorescent imaging and quantitative image analysis demonstrated that repetitive mild head impacts led to an increase in BBB permeability in both the second motor and orbital cortex (Figures 5A,B,D). This is highlighted by increased dextran accumulation in the brain parenchyma (white arrows) as well as within vascular cells, potentially suggesting increased retention by endothelial cells. In contrast, low levels of parenchymal and vascular dextran were observed in the substantia nigra (Figures 5C,D). These results agree with QUTE-CE results.

## Discussion

The present study followed the immediate and accumulative effects of one, two, and three mild head impacts on site-specific changes in BBB permeability over the entire brain in rats both at the individual and group levels. At the group level, only a modest effect was displayed after one hit, and the consequences of a second and third hit were more severe, affecting a greater area of the brain. These results are discussed with respect to the clinical implications of mild repetitive head impacts without structural brain damage and the use of a novel imaging modality, QUTE-CE MRI, to identify these putative changes in injury-induced permeability.

There are numerous studies reporting moderate to severe traumatic brain injury (TBI) can have long-term deleterious effects on BBB integrity and function (18). There are numerous human and animal studies using various *in-vivo* and *in-vitro* methods showing that repetitive mild TBI separated by short intervals of time posse a significant risk to the brain and vulnerability to neurodegenerative disorders with aging (45–48). Indeed, the cerebral vascular injury from TBI impacts the function of the neurovascular unit and may be a contributing factor in the pathophysiology of neurodegenerative diseases (49, 50). Indeed, moderate to severe TBI is a risk factor for Alzheimer's (51, 52), Parkinson's (53) and Amyotrophic Lateral Sclerosis (54). However, mild head injury is the most prevalent form of head injury and is estimated to affect over 42 million people worldwide every year due mostly to falls and motor accidents (1, 7). Repetitive mild head impacts in organized sports or on the battlefield have raised concerns about their long-term effects on neurodegeneration, e.g., CTE (9). The health community lists guidelines for diagnosing these mild head injuries that include self-reports of transient confusion, disorientation, impaired consciousness, or dysfunction in memory around the time of the injury and, importantly, no apparent structural damage as determined with imaging (1, 55, 56). Unfortunately, many existing models for TBI in animals are far too severe and therefore do not mimic the more insidious effect of mild impact.

In our model, the skull was intact for all animals, and there was no radiological evidence of a contusion, or structural damage, but only edema near the site of impact. There are several studies on the neurobiological effects of single and repetitive head impacts in closed head models—these are not considered TBI because there is no structural brain damage. For example, Kane and coworkers assessed the effects of repetitive mild head impacts on over 300 mice using measures of behavior and postmortem histology (19). With a closed head momentum exchange model, they delivered a single impact over five consecutive days. The rate of mortality after multiple hits was ca 10%. Minor deficits in balance and motor coordination recovered over time. There was no brain edema as determined by the wet/dry method to assess changes in water content. The cortex under the site of impact showed no staining for IgG suggesting there was no increase in BBB permeability. Reactive gliosis was observed in the cortex and hippocampus. Logsdon et al., reported two mild blast injuries, 12–15 min apart, caused an immediate increase in BBB permeability to radiolabeled albumin across much of the brain (21). The change in BBB permeability was biphasic, resolving within 24 h but reappearing 72 h later. The delayed response was region-specific, affecting the frontal cortex, hippocampus, thalamus, and medulla. The immediate and latent changes in BBB permeability could be explained by a reduction in the brain endothelial cell expression of tight junction protein, claudin-5. Ren, and colleagues, developed a method (“hit and run”) for giving a *single mild head impact* to mice without structural brain damage (20). There were no differences between sham and impacted mice for measures of cognitive behavior but there was a decrease in motor performance on the rotarod that persisted for 28 days. There was compelling evidence of diffuse gliosis with white matter degeneration that persists for 7–14 days. However, there was no edema or appreciable change in BBB permeability as measured by Evans Blue extravasation. Tagge et al. published a comprehensive study in over 300 mice on mild repetitive head injury using a closed head momentum exchange model in *fully awake* mice (22). Two head impacts separated by 15 min, were followed by a battery of neurobehavioral tests, imaging, and histology for BBB permeability. There was no evidence of structural brain damage. After one impact, ca 82% of these mice (*n* = 166/203) exhibited minimal neurological impairment. Of these mice, 66.5% showed minimal or no impairment after the 2nd head impact. All animals recovered with 3 h. At 24 h there was sparse gliosis. However, reactive astrocytosis was observed at 3 days and 2 weeks post injury characterized by ramified processes and swollen endfeet around small blood vessels at the cortical site of impact. Microgliosis appeared at 3 days but was mostly resolved by 2 weeks. This time course of neuropathology was also reported in *mice hit only once*. Fifty percentage of the mice showed no change in BBB permeability, while 40% showed minimal brain damage marked only by Evans blue extravasation. BBB permeability at the site of impact was noted within 3 h and persisted for 3 days. Pham et al. reported a *single mild head* impact in *awake rats*, again without structural brain damage, induced microgliosis at 1-, 3-, and 14-days post insult around impact and astrocytosis at day 3 in the hippocampus (57).

Measures of BBB permeability are variable across and within the different models described above. These studies on repetitive mild head impacts in mice, using different methods to inflict injury, all without structural brain damage, speak to the variability in behavioral and neurobiological outcomes. Judging these types of mild head impacts by changes in behavior would seem uninformative given the absence and/or transience of such measures across different models. The neuropathology, particularly gliosis would appear to offer a better biomarker as it is consistent in all models. The absence of any change in permeability with mild head impact may be due to the timing of the observation. Histological validation of changes in BBB permeability is a snapshot of a single time point of a dynamic process that is biphasic as described by Logsdon et al. (21), with an onset and duration noted by Tagge et al. (22). In a just published study using apparent diffusion coefficient from diffusion weighted imaging as a proxy of vasogenic edema from disruption in BBB permeability, we reported increased edema that peaked at 6 h post one mild head impact that resolved within 24 h (58). Hence post-mortem histology for assessing BBB permeability has its limitations set by the time from insult to animal sacrifice.

Dynamic contrast enhanced (DCE) MRI with gadolinium-based contrast agents (GBCAs) is a method for assessing brain vascular permeability. However, the inaccuracy of DCE MRI is the major drawback. DCE looks at the rate of exchange of vascular contrast agent between plasma and the extracellular space of the brain parenchyma generating a volume transfer constant K_Trans_ (26). DCE MRI has been used to measure BBB permeability following moderate TBI in rodents (59–61). In humans, DCE MRI shows an increase in BBB permeability in response to moderate to severe TBI around damage tissue within 24 h of injury that persists for days (62). DCE MRI has also been used to evaluate vascular permeability in mild head injury. Athletes playing American football and thought to have mild, sub-concussive head injuries show evidence of increased BBB permeability (63). Using DCE-MRI, O'Keeffe and colleagues reported BBB disruption in response to repetitive mild concussions in rugby players after a season and mixed martial arts fighters within days after a competitive fight (64). In a just published study by Veksler et al., using a modified DCE MRI approach, they were able to generate maps of increased BBB permeability in American football players vs. controls (i.e., athletes from non-contact sports) that persisted for months after contact (65). The permeability maps were region specific, but primarily correlated with changes in white matter tracts. The permeability was characterized by a slow blood-to-brain transcellular transport mechanism underlying chronic BBB dysfunction. More recently, higher accuracy measurements have been achieved in rodents (31, 32) and humans (33, 34), however DCE-MRI remains limited to 2-dimensional imaging with thick slabs (1 mm in mice, 5 mm in humans)—in addition to requiring GBCAs.

QUTE-CE MRI was developed to generate quantitative vascular biomarkers for precision medicine (35–39). Ferumoxytol MRI with optimized 3D Ultra-Short Time-to-echo (UTE) pulse sequences produces angiographic images unparalleled to time-of-flight imaging or GBCA first-pass imaging (see Figure 1, Supplementary Data). The contrast agent ferumoxytol, is an ultra-small superparamagnetic iron oxide nanoparticle (USPION) with a dextran coating. Since the size exceeds the cutoff (~6 nm) for glomerular filtration, ferumoxytol is not cleared by the kidney, and instead is an excellent blood pool contrast agent with a long intravascular half-life of ~15 h (66). However, ferumoxytol has received an FDA black box warning for rare, but serious anaphylactic reactions. In clinical practice, the risk for anaphylaxis is now mitigated by administrating the agent over a 10–15-min infusion period—rather than a bolus (67). Nevertheless, development of an FDA approved hypoallergenic USPION formulation would be greatly beneficial for the safest clinical implementation of QUTE-CE MRI.

This model of repetitive head impact combined with non-invasive QUTE-CE enabled us to investigate two clinically relevant questions: (1) Does a single mild impact to the head compromise BBB integrity? (2) Do several mild impacts, within days of one another, exacerbate the risk of BBB injury? The question of whether the site-specific changes in BBB permeability would resolve over time or persist and evolve into cerebral vascular injury is unknown but could be followed by end-of-life studies using non-invasive imaging. In a recently published study using the three, mild head impact model described here, we reported alterations in indices of anisotropy reflecting white and gray matter damage and loss of functional connectivity between various brain regions 7–8 weeks post insult (43).

The imaging data confirmed that a single hit, did indeed, increase BBB permeability and that repeated impacts broadened the area of vulnerability to distal regions of the brain. The sensitivity of QUTE-CE to resolve insult in individual animals revealed the variability between subjects both in sensitivity to head impact and site-specific vulnerability. At baseline, all rats exhibited minimal leakage as measured with the method. While all rats were impacted on the dorsal cranium in the region of the forebrain, individual responses varied. Some rats were more resilient to head impact, as in the case of Rat 5 who responded primarily only after the 3rd impact, while others like Rats 1, 2, and 4 showed widespread increases in BBB permeability after a single head impact. All the head impacts included increased BBB permeability in the olfactory system and striatum, but injury to hindbrain areas was less predictable. Only after the 3rd impact was the hippocampus, CA1 and subiculum, affected. Each rat responded differently, an observation that in many ways reflects the human experience.

## Limitations

This was an exploratory study to determine whether QUTE-CE imaging was sensitive enough to pick up changes in BBB permeability to mild head impacts, and as such, had several limitations. Foremost was a comparison to DCE the standard method used in the clinic to evaluate changes in BBB permeability, including examination of BBB leakage of nanoparticles versus macromolecular GBCAs. These studies were unfunded and exploratory and as such we did not have the resources to make a head-to-head comparison between QUTE-CE and DCE. Also lacking in these studies was the use of females. We are aware of the many studies reporting sex differences in response to TBI (68–73). Recent reviews of the literature, report male/female differences in morbidity and mortality following TBI (74, 75). Also, the histology was only focused on a single fluorescent marker to show BBB permeability. Staining for markers of gliosis, neuron viability and capillary integrity would have provided a better understanding of the effect on mild head impact on the neurovascular unit. Lastly, it would have been interesting to know how long between impacts is necessary to avoid the compounded effect of multiple insults or the potential confound of CBV changes in tissue due to insult. This window of vulnerability was tested with intervals of 24 h only. Would 1 week or longer between impacts have negated the exacerbated increase in BBB permeability?

## Summary

Quantitative measures of increased BBB permeability with QUTE-CE MRI appears to be feasible, even in individual subjects, as assessed in this exploratory study with 5 animals. At baseline, minimal leakage was present, and multiple hits exacerbated BBB leakage, which appeared to peak after the second hit. QUTE-CE may thus be a method to non-invasively follow the consequences of mild head injury at the level of the microvasculature in individual subjects. Given the reports that BBB leakage may play a critical role in the pathophysiology of early AD (76) and cerebral amyloid angiopathy (77) QUTE-CE may provide a sensitive method for early diagnosis and assement of treatment efficacy.

## Data Availability Statement

The original contributions presented in the study are included in the article/Supplementary Material, further inquiries can be directed to the corresponding author/s.

## Ethics Statement

The animal study was reviewed and approved by IACUC Northeastern University.

## Author Contributions

CF, PK, EE, and CG concept, drafting, and interpretation. JQ, PK, JL, and IH execution and analysis. All authors have contributed substantially to the manuscript and read and agreed to the published version of the manuscript.

## Funding

This work was supported by an AHA pre-doctoral fellowship (18PRE33960461), NIH grant (K01-HL125499), HHMI funded Inclusive Excellence Award to Northeastern University (PI Ondrechen) and Imaginostics.

## Conflict of Interest

CF has a financial interest in Animal Imaging Research, the company that makes the RF electronics and holders for animal imaging. CF, CG, and PK are inventors of QUTE-CE MRI related patents and CG is CEO of Imaginostics, a company created to translate the QUTE-CE MRI technology to the clinic. The remaining authors declare that the research was conducted in the absence of any commercial or financial relationships that could be construed as a potential conflict of interest.

## Publisher's Note

All claims expressed in this article are solely those of the authors and do not necessarily represent those of their affiliated organizations, or those of the publisher, the editors and the reviewers. Any product that may be evaluated in this article, or claim that may be made by its manufacturer, is not guaranteed or endorsed by the publisher.
