# Supplementary material for: Quantitative Imaging of Blood-Brain Barrier Permeability Following Repetitive Mild Head Impacts
Source: Front Neurol. 2021 Sep 30;12:729464. doi: 10.3389/fneur.2021.729464 (PMC8515019; doi:10.3389/fneur.2021.729464)
Supplement: Supplementary file 1 [file Image_1.pdf]

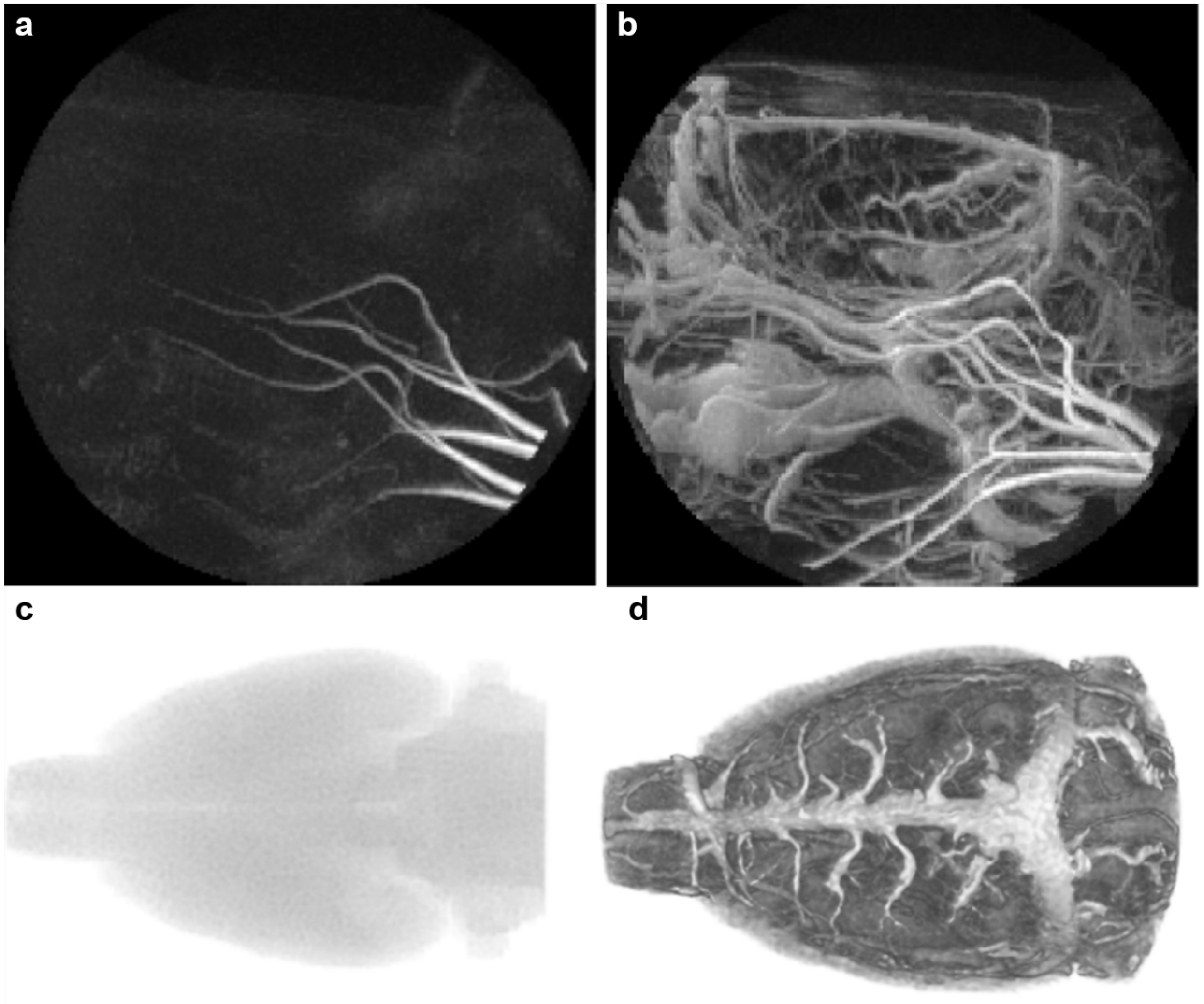

Supplementary Figure 1. QUTE-CE MRI isolated vascular compartments. (a) pre- and (b) post-contrast ferumoxytol (14mg/kg) sagittal Maximum Intensity Projection (MIP) images of the whole rat head. (c-d) The corresponding segmented images of the brain, with the large vessel on the dorsal surface being the superior sagittal sinus.
