# Supplementary material for: Quantitative Imaging of Blood-Brain Barrier Permeability Following Repetitive Mild Head Impacts
Source: Front Neurol. 2021 Sep 30;12:729464. doi: 10.3389/fneur.2021.729464 (PMC8515019; doi:10.3389/fneur.2021.729464)
Supplement: Supplementary file 2 [file Image_2.pdf]

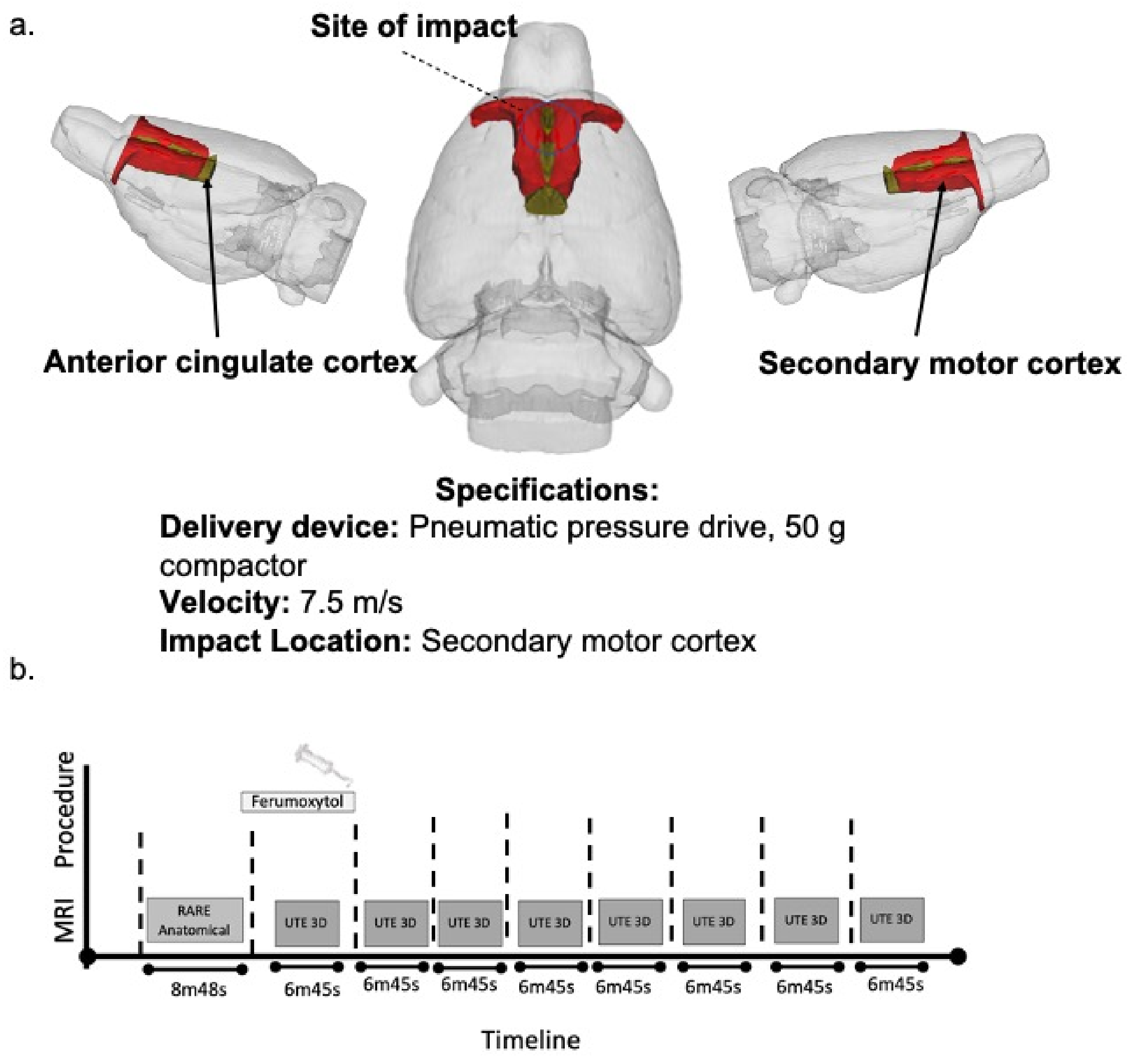

Supplementary Figure 2. MRI scan procedures. (a) The site of the head impact is displayed in the image. Note that the animal was under 5% isoflurane prior to head impact. (b) The scan procedure is displayed here, which was performed within 1 hour of head impact. Procedure consisted of an anatomical scan, pre-contrast UTE, ferumoxytol infusion, and seven more post-contrast UTE scans.
