# Supplementary material for: Quantitative Imaging of Blood-Brain Barrier Permeability Following Repetitive Mild Head Impacts
Source: Front Neurol. 2021 Sep 30;12:729464. doi: 10.3389/fneur.2021.729464 (PMC8515019; doi:10.3389/fneur.2021.729464)
Supplement: Supplementary file 3 [file Image_3.pdf]

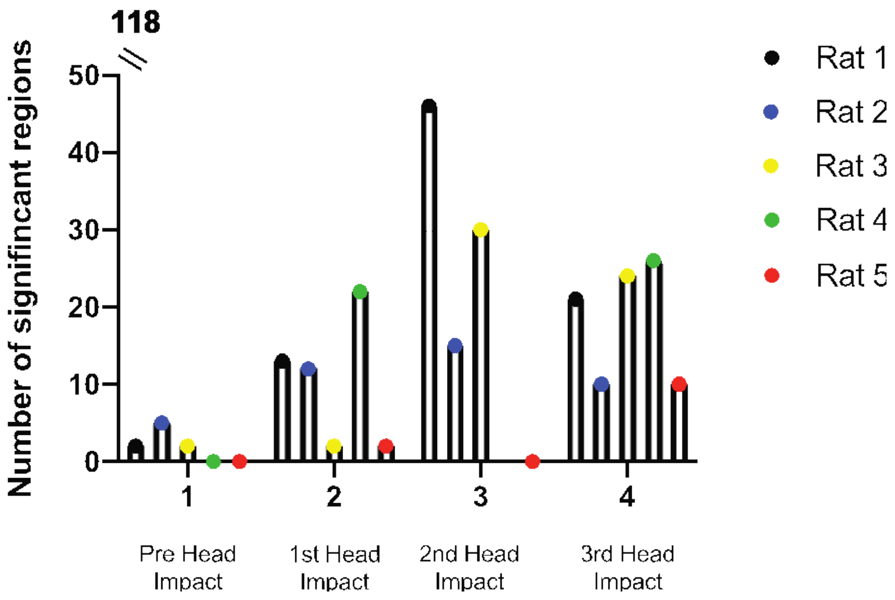

Supplementary Figure 3. Number of significant regions per head impact. The number of brain regions for each individual animal that was significant is displayed per scan session. Significance was determined with a 90% confidence interval, followed by a Bonferroni correction.
