# Supplementary material for: Quantitative Imaging of Blood-Brain Barrier Permeability Following Repetitive Mild Head Impacts
Source: Front Neurol. 2021 Sep 30;12:729464. doi: 10.3389/fneur.2021.729464 (PMC8515019; doi:10.3389/fneur.2021.729464)
Supplement: Supplementary file 4 [file Image_4.pdf]

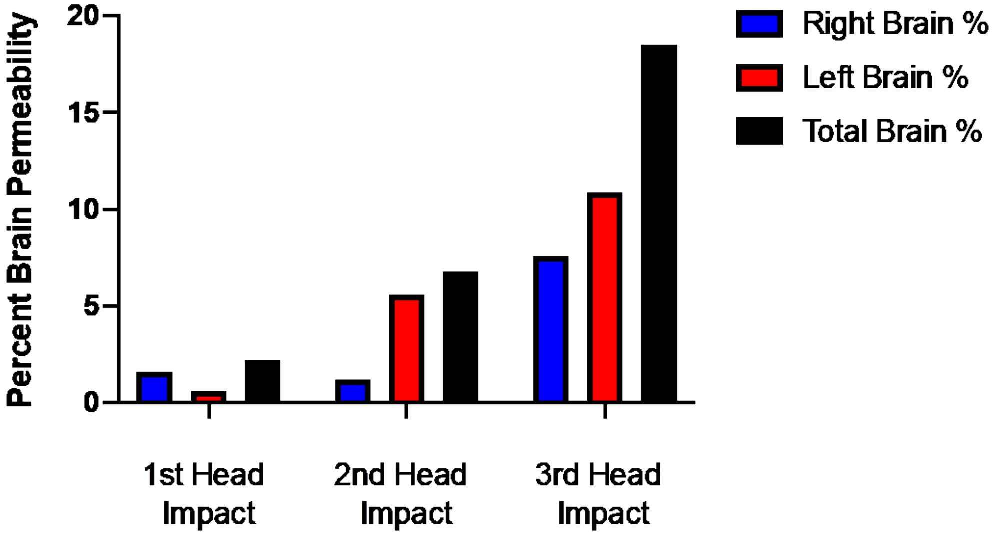

Supplementary Figure 4. Bilaterality **of increased brain permeability from baseline.** Total brain volume significant for increased BBB permeability as calculated with ANOVA, followed by a Benjamin Hochberg correction($p<0.05$; FDR=0.1).
